# An implementation study of oral and blood‐based HIV self‐testing and linkage to care among men in rural and peri‐urban KwaZulu‐Natal, South Africa

**DOI:** 10.1002/jia2.25514

**Published:** 2020-06-26

**Authors:** Adrienne E Shapiro, Alastair van Heerden, Meighan Krows, Kombi Sausi, Nsika Sithole, Torin T Schaafsma, Olivier Koole, Heidi van Rooyen, Connie L Celum, Ruanne V Barnabas

**Affiliations:** ^1^ Department of Global Health University of Washington Seattle USA; ^2^ Department of Medicine Division of Infectious Diseases University of Washington Seattle USA; ^3^ Human Sciences Research Council Sweetwaters South Africa; ^4^ MRC/Wits Developmental Pathways for Health Research Unit (DPHRU) University of the Witwatersrand Johannesburg‐Braamfontein South Africa; ^5^ Africa Health Research Institute Mtubatuba South Africa; ^6^ London School of Hygiene and Tropical Medicine London United Kingdom

**Keywords:** HIV infections, male, workplace, South Africa, mass screening, serologic tests

## Abstract

**Introduction:**

In South Africa, HIV‐infected men are less likely than women to test and know their status (the first UNAIDS “90‐90‐90” target), and men have worse outcomes across the HIV care cascade. HIV self‐testing (HIVST) may address this testing disparity but questions remain over the most effective distribution strategy and linkage following a positive test result. We implemented a men‐focused HIVST distribution programme to evaluate components contributing to participation and retention.

**Methods:**

We conducted an implementation study of multi‐venue HIVST kit distribution in rural and peri‐urban KwaZulu‐Natal (KZN), South Africa. We distributed HIVST kits at community points, workplaces and social venues for on site or take‐home use. Clients could choose blood‐based or oral‐fluid‐based HIVST kits and elect to watch an in‐person or video demonstration. We provided a USD2 incentive to facilitate reporting test results by phone or SMS. Persons with reactive HIVST results were provided immediate confirmatory tests (if used HIVST on site) or were referred for confirmatory testing (if took HIVST off site) and linkage to care for ART initiation. We describe the testing and linkage cascade in this sample and describe predictors of reactive HIVST results and linkage.

**Results:**

Between July and November 2018, we distributed 4496 HIVST kits in two regions of KZN (96% to men, median age 28 (IQR 23 to 35). Most participants (58%) chose blood‐based HIVST and 42% chose oral‐swab kits. 11% of men were testing for the first time. A total of 3902 (83%) of testers reported their test result to the study team, with 314 (8%) screening positive for HIV. Among 274 men with reactive HIVST results, 68% linked to ART; no significant predictors of linkage were identified. 10% of kit users reported they would prefer a different type (oral vs. blood) of kit for repeat testing than the type they used.

**Conclusions:**

HIVST is acceptable to men and rapid distribution with optional testing support is feasible in rural and peri‐urban settings. HIVST kits successfully reached younger men and identified undetected infections. Both oral and blood‐based HIVST were selected. Scaling up HIVST distribution and guidance may increase the number of first‐time testers among men and help achieve the first UNAIDS “90” for men in South Africa.

## 1 INTRODUCTION

In sub‐Saharan Africa, men access HIV testing and treatment services at lower rates than women [1, 2]. Across the UNAIDS 90‐90‐90 cascade for HIV testing, taking ART and virological suppression on ART, men have worse indices than women [1, 3]. Following the WHO’s 2016 recommendation to provide HIV self‐test (HIVST) services to improve access to testing, prevention and treatment, South Africa introduced guidelines for HIVST services and support in 2017 [4, 5, 6]. In South Africa, the testing gap between men and women has persisted and men have not met any 90‐90‐90 targets, despite rapid scale‐up of antiretroviral therapy (ART) access and adoption of universal test‐and‐treat policies [7, 8]. Increasing access to HIVST through community‐based distribution of kits may overcome some barriers men experience to learning their HIV status, including wanting to avoid female‐dominated clinic settings, by providing the convenience to test at home or during hours compatible with a work schedule, overcoming traditional masculine ideals in this population about care‐seeking and self‐reliance, and reducing initial perceived stigma associated with accessing clinic settings [9, 10, 11, 12, 13].

Early, large‐scale demonstrations of HIVST kit distribution showed success in increasing HIV testing among men in three sub‐Saharan African countries using approaches including community‐based, workplace‐based and health facility‐based distribution [14]. Studies of HIVST distribution to men by female partners have demonstrated moderate linkage to care [15, 16], but these studies often relied on secondary reporting about men’s behaviour by female partners. Less is known about successful linkage for men after a positive HIV self‐test received in a community programme. We conducted a multi‐venue distribution programme of HIVST kits targeting men in the KwaZulu‐Natal Province of South Africa. Our aim was to understand whether HIVST distribution is feasible to engage men in testing, to determine the yield of HIV detection and linkage to care for men by providing HIVST in South African communities and to determine predictors of retention along the HIV cascade for men who use HIVST, in order to better optimize engagement for men.

## 2 METHODS

We conducted and evaluated a multi‐venue distribution programme of HIVST kits in peri‐urban and rural districts of KwaZulu‐Natal, South Africa, that targeted adult men. In order to understand the impact of HIVST in resource‐limited settings with a view towards programmatic scale up, we designed the distribution to be pragmatically implemented, with lay staff providing most services and minimal participation incentives.

### 2.1 HIVST kit distribution procedures

**Figure 1:**
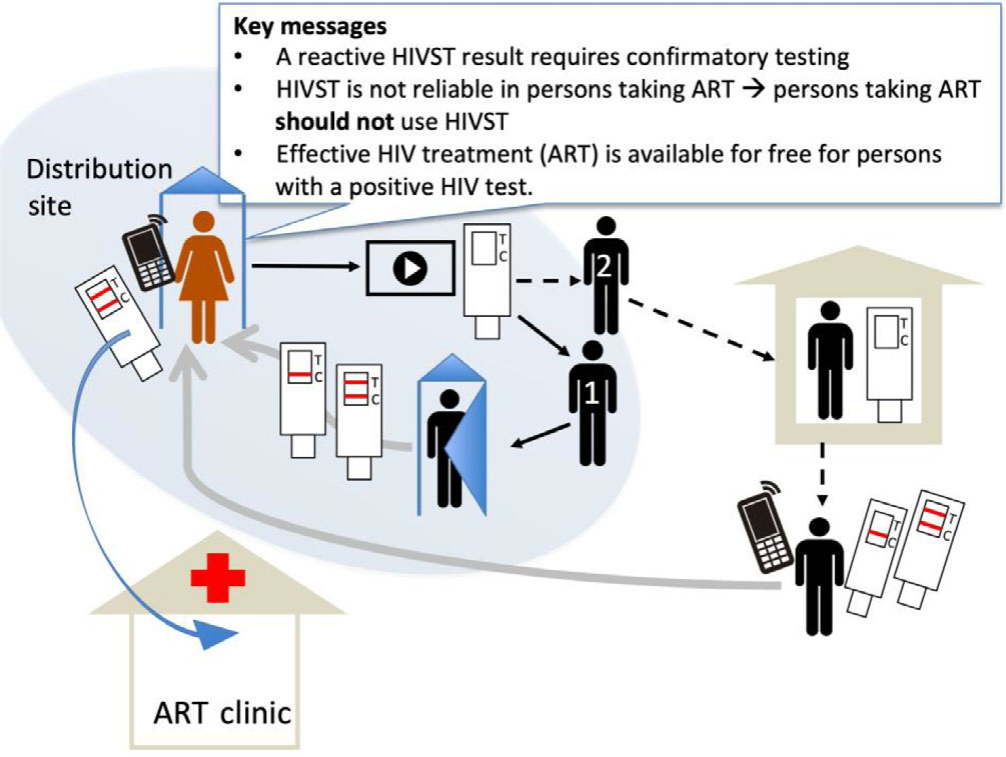
Schematic diagram of HIVST distribution and testing procedures. At the distribution site, study staff provided education, distributed HIVST kits and demonstrated or showed videos of how to use kits. 1) On site testing (solid black arrows): men received a test kit, used the kit in a private booth, then reported their result (solid grey arrow) to study counselling staff. Counselling staff referred to local clinics (blue arrow) for ART initiation if the HIVST was positive and confirmed. 2) Off site testing (dashed arrows): Men collected a kit at the distribution site, watched a demonstration, then took the kit home. After using the kit, they would call or SMS to study staff to report results (solid grey arrow), receive counselling and referral to clinic for confirmatory testing & ART initiation.

Teams of 3‐6 lay counsellors set up distribution sites (4‐8 events per week during the study period) in community centres, at male‐dominated workplaces (farms, agricultural industry, factories, construction sites, taxi ranks), and at venues including sporting events, tuckshops, bottle shops and other public gatherings. Two types of HIVST kits were available at distribution events: a fingerprick blood‐based kit (iTest, Atomo, Sydney, Australia)[17] and an oral‐fluid‐based kit (Oraquick, OraSure, Bethlehem, PA, USA). At each event, counsellors provided a brief informational session on the importance of testing for HIV, what HIVST is, and emphasized key messages about HIVST (Figure 1). Counsellors conducted a live demonstration of how to use and interpret both test kits, and provided cell‐phone videos demonstrating kit use, available for watching on site or at home. Men were then invited to select a test kit. They had the option to use the test immediately in a private booth, with or without assistance from a staff person, or take a kit for use after the event. Event duration ranged from one hour workplace sessions to half‐day community sessions, with small group information sessions repeated throughout an afternoon.

### 2.2 Data collection and ascertaining results

Counsellors obtained informed consent from men interested in participating prior to distributing kits. The only eligibility criterion was being age 18 or older. Participants completed a baseline questionnaire about demographics, HIV testing history, sexual behaviours, alcohol use, test kit preferences and cell phone number. They received a test kit labelled with a study identification number.

Participants were asked to report the results of their HIVST to the study team. All participants received a callback card with their HIVST kit to report the results of their self‐test to the study team, as well as a voucher for cellphone airtime (valued at USD2) to be redeemed at the time of reporting results.. Participants who tested on site presented to study staff after testing to voluntarily report their results or could elect to leave the site and report results later using the callback card. Participants who were directly assisted by study staff in conducting the HIVST reported their results directly as they received them. Persons taking kits off site reported results by calling or sending an SMS/text message to the number on the callback card for a free callback by a study staff person. Staff then contacted the participant and ask for the HIVST result. Study staff provided post‐HIVST counselling including referral for confirmatory testing and ART if the HIVST was reactive, or HIV prevention information and referral for voluntary male medical circumcision if the HIVST was nonreactive.

Participants received a non‐identifying reminder text message after 2 weeks if they had not yet reported their result. (“Act now‐‐test for HIV! Did the test? Call or send a Please Call Me to XXX.”). The reminder was repeated at 1 and 2 months. If no result was reported by 2 months, staff made an outreach call to assess test use and results.

After participants reported their results (either in person or by phone) and received counselling and referrals, staff distributed the airtime incentive and administered a brief questionnaire to assess experience, usability, acceptability and preferences about HIVST.

Distribution events also provided confirmatory HIV testing on site (using standard rapid tests performed by lay counsellors trained in the national Department of Health algorithm) and linkage to ART for persons with positive confirmatory tests. Linkage was offered either through referral to a local clinic of the participant’s choice or referral into an ongoing ART delivery study (DO ART study, ClinicalTrials.gov NCT02929992).

### 2.3 Ascertaining linkage to care and prevention

All participants who reported their HIVST results received a phone call 2 months after results reporting to assess whether they had linked to confirmatory testing and ART or preventive services. We defined linkage to ART as attending clinic (or enrolling in the DO ART study) and initiating ART. Study staff validated a portion of self‐reported ART initiation by confirming registration with ART clinics.

### 2.4 Statistical analysis

Percentages were calculated for descriptive statistics. Crude and adjusted relative risks of positive HIVST results were calculated among men with a known HIVST result for selected predictors. Crude and adjusted relative risks of linkage to care were calculated among men with a reactive HIVST. Relative risks were, adjusted for potential sociodemographic confounders: age, study site, testing venue type, testing location, HIV testing history, circumcision status, educational status, marital status, employment status and self‐reported alcohol use. Adjusted relative risks were calculated in R using modified Poisson regression for binary outcomes with robust variance estimation.

### 2.5 Ethics

The study was reviewed and approved by the institutional review boards or ethics committees of the HSRC, University of KwaZulu‐Natal, and the University of Washington. All participants provided informed consent.

## 3 RESULTS

### 3.1 Characteristics of tests distributed and recipients

Between July and November 2018, study teams distributed 4496 HIVST kits (1890 oral fluid kits, 2605 blood‐based kits) to South African adults in two districts.

Persons receiving HIVST kits at the distribution sites were overwhelmingly men (96%) by design (Table 1). The median age was 28 years (IQR 23 to 35). Over a third (36%) of participants were unemployed. Eleven percent of participants reported never testing for HIV and 40% of previous testers reported they last tested more than 12 months earlier. Fifty‐four percent of men reported being circumcised at baseline. Most (4216, 94%) were unmarried and 37% reported having more than one current sexual partner. More than half of participants reported some alcohol use.

**Table 1:** Characteristics of HIVST kit distribution and recipients

	Total N (%)
Study district	
Peri‐urban	1863 (41%)
Rural	2632 (59%)
Distribution setting	
Mobile van	3175 (71%)
Social venue‐based	211 (5%)
Workplace	1003 (22%)
Other	107 (2%)
Kit type selected	
Oral fluid	1890 (42%)
Blood‐based	2605 (58%)
Age, median (IQR)	28 (23 to 35)
Sex	
Male	4307 (96%)
Female	189 (4%)
Education	
Primary	572 (13%)
Secondary+	3897 (87%)
Unemployed	1626 (36%)
Marital status	
Married	280 (6%)
Unmarried	4216 (94%)
Number of current sex partners	
0	144 (3%)
1	2697 (60%)
>1	1636 (37%)
Ever tested for HIV	
Yes	3982 (89%)
No	505 (11%)
Circumcised (men)	
Yes	2407 (56%)
N	1879 (44%)
Alcohol use (drinks in past week)	
0	1865 (41%)
1 to 6	1943 (43%)
7+	665 (15%)

IQR, inter‐quartile range.

Almost all men (86%) stated that the reason they elected to test using HIVST was that they wanted to know their HIV status. Seven percent stated their reason was HIVST was more convenient than facility‐based HIV counselling and testing, and 3% stated they participated to receive the airtime incentive.

### 3.2 HIVST results and linkage

Test results were reported for 3486 (81%) of HIVST kits distributed to men. Overall, a total of 8% of reported test results were reactive for HIV antibodies. Among 274 men with reactive HIVST results, linkage outcome was determined for 81% (the remainder could not be reached after multiple attempts, or confirmed remotely); 72% of men with reactive HIVSTs received a confirmatory test (6 confirmatory tests were negative), and 95% of men with a positive confirmatory test linked to care and initiated ART. Overall, 68% of men with reported reactive HIVST results successfully linked to ART.

**Figure 2:**
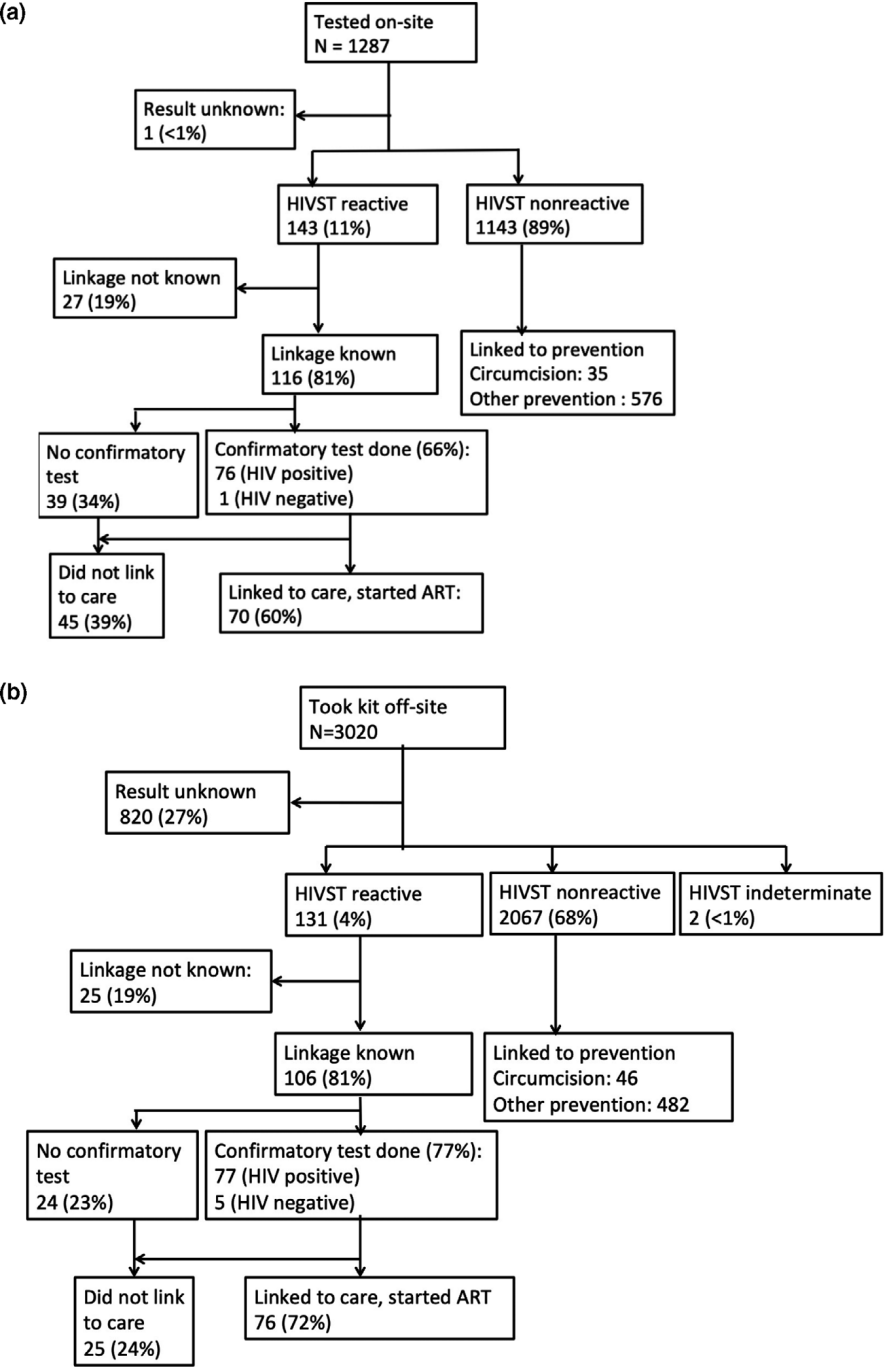
HIVST testing, results and linkage cascade for N = 4307 men in South Africa given an HIVST kit. **(A)** Men who tested at the distribution site (on site) using HIVST kit; **(B)** Men who took HIVST kit off site.

Retention along the cascade differed between men who tested on site and men who took kits off site to test (Figure 2). Results were ascertained for 100% of tests used by men testing on site; 11% were reactive (Figure 2A). Among men with reactive tests whose linkage status was confirmed (81%), 60% linked to care for confirmatory testing and initiating ART. In comparison, 73% of men who tested off site reported HIVST results, 4% were reactive, linkage was confirmed for 81% and 72% linked to care and started ART (Figure 2B). Participants who did not report HIVST results did not differ significantly from participants with known results with regards to any baseline characteristics assessed, except for the kit type selected. Participants with unknown results were slightly more likely to have chosen a blood‐based test (472/745, 63%) compared to participants with known results (2133/3750, 57%).

We evaluated predictors of a reactive HIVST among men whose test results were known (Table 2).

**Table 2:** Predictors of reactive HIVST results in men

Predictor	Categories	n/N	Crude Risk Ratio	95% Confidence interval *p*‐value	Adjusted Risk Ratio^a^	95% Confidence Interval *p*‐value
Age	<30 >=30	84/ 1993 190/ 1491	‐ref‐ 3.02	2.34 to 3.91 *p* < 0.0001	‐ref‐ 2.28	1.74 to 2.99 *p* < 0.0001
Self‐test location	On site Off site	143/ 1286 131/ 2198	‐ref‐ 0.54	0.42 to 0.68 *p* < 0.0001	‐ref‐ 0.66	0.49 to 0.88 *p* = 0.0044
Ever tested for HIV before	No Yes	55/ 379 219/ 3097	‐ref‐ 0.49	0.36 to 0.65 *p* < 0.0001	‐ref‐ 0.70	0.51 to 0.95 *p* = 0.023
Education level	Primary Secondary +	55/ 416 215/ 3049	‐ref‐ 0.53	0.40 to 0.72 *p* < 0.0001	‐ref‐ 0.84	0.61 to 1.16 *p* = 0.29
Employment	Unemployed Employed	108/ 1258 166/ 2226	‐ref‐ 0.87	0.68 to 1.11 *p* = 0.25	‐ref‐ 0.86	0.65 to 1.14 *p* = 0.29
Marital Status	Married Not married	17/ 208 257/ 3276	‐ref‐ 0.96	0.59 to 1.57 *p* = 0.87	‐ref‐ 1.67	1.00 to 2.77 *p* = 0.049
Alcohol use (drinks/week)	0 to 6 >=7	199/ 2956 75/ 528	‐ref‐ 2.11	1.62 to 2.75 *p* < 0.0001	‐ref‐ 1.72	1.31 to 2.26 *p* < 0.0001
Circumcised	No Yes	188/ 1514 83/ 1955	‐ref‐ 0.34	0.26 to 0.44 *p* < 0.001	‐ref‐ 0.43	0.33 to 0.57 *p* < 0.0001

^a^ Adjusted for: test site, age, self‐test location, ever tested for HIV before, education level, employment status, marital status, alcohol use and circumcision status.

Men who took an HIVST kit off site to test had a lower risk of a reactive HIVST compared to men who tested on site (adjusted risk ratio 0.66, 95% CI 0.49 to 0.88). Factors associated with increased risks of a reactive HIVST included older age, uncircumcised status and unmarried status. Self‐reported alcohol use of seven drinks or more per week was associated with 72% higher risk of a reactive HIVST compared to less alcohol use (aRR: 1.72, 95% CI 1.31 to 2.26). Men who reported previously testing for HIV had a decreased risk of a reactive HIVST (aRR 0.70, 95% CI 0.51 to 0.95), compared to men who reported being first‐time testers.

Men testing off site were slightly more likely to link to care than men testing on site, (RR 1.24, 95% CI 0.89 to 1.71), but this was not statistically significant. The increased likelihood disappeared after adjusting for demographic features (aRR 1.04, 95% CI 0.75 to 1.63); no statistically significant predictors of linkage to ART were found among men with reactive HIVST results with linkage data available (Table 3). Men with reactive results who had not linked to care at the time of the linkage ascertainment call cited several reasons, including lack of time to go to clinic, having to be at work during the hours that the clinic is open, and being concerned about long wait times at clinic.

**Table 3:** Predictors of linkage to ART among men with reactive HIVST results

Predictor	Categories	n/N	Crude Risk Ratio	95% Confidence interval *p*‐value	Adjusted Risk Ratio^a^	95% Confidence Interval *p*‐value
Age	<30 >=30	39/57 107/159	‐ref‐ 0.98	0.68 to 1.42 *p* = 0.93	‐ref‐ 1.11	0.75 to 1.63 *p* = 0.60
Self‐test location	On site Off site	70/115 76/101	‐ref‐ 1.24	0.89 to 1.71 *p* = 0.2	‐ref‐ 1.04	0.72 to 1.52 0.83
Ever tested for HIV before	No Yes	32/49 114/167	‐ref‐ 1.05	0.71 to 1.55 *p* = 0.82	‐ref‐ 1.01	0.67 to 1.53 *p* = 0.96
Education level	Primary Secondary +	31/43 113/171	‐ref‐ 0.92	0.62 to 1.36 *p* = 0.67	‐ref‐ 0.96	0.62 to 1.47 *p* = 0.84
Employment	Unemployed Employed	61/80 85/136	‐ref‐ 0.82	0.59 to 1.14 *p* = 0.24	‐ref‐ 0.86	0.59 to 1.25 *p* = 0.42
Marital Status	Married Not married	10/14 136/202	‐ref‐ 0.94	0.50 to 1.79 *p* = 0.86	‐ref‐ 0.87	0.44 to 1.73 *p* = 0.69
Alcohol use (drinks/week)	0 to 6 >=7	111/161 35/55	‐ref‐ 0.92	0.63 to 1.35 *p* = 0.68	‐ref‐ 0.95	0.64 to 1.40 *p* = 0.79
Circumcised	No Yes	105/157 41/57	‐ref‐ 1.08	0.75 to 1.54 *p* = 0.69	‐ref‐ 1.17	0.8 to 1.71 *p* = 0.42

^a^ Adjusted for: age, self‐test location, ever tested for HIV before, education level, employment status, marital status, alcohol use and circumcision status.

### 3.3 Experiences and preferences

Men reporting HIVST results (N = 3488) completed a questionnaire about their experience using the kits and learning their HIV status. Most (3389, 97%) reported that using the test kit was “easy” or “very easy.” Most men (2334, 67%) reported that they learned to use the kit from the in‐person demonstration, and 2937 (84%) reported that they did not require additional help in using the kit. 697 (20%) reported receiving help from a study counsellor using the kit or interpreting results and 26 (1%) requested help from a friend or family member.

Men were asked what type of HIVST kit (blood‐ or oral‐fluid‐based) they would prefer to use in the future, based on their experience with the type they used. Over 90% of men reported a preference to use the same type they had used initially: 1428 (90%) of Oraquick users reported preferring to do a repeat test with Oraquick, and 1928 (93%) of the blood‐based iTest users stated they would prefer to use the iTest again.

At the two‐month outreach call to ascertain HIVST results, 92/3576 (2.6%) men reported they did not use the kit they received. The main reasons cited for not using the HIVST were: being unsure how to use it, not having time to use it, and losing the kit. Five men stated that they attempted to use the blood‐based kit and could not obtain blood with the lancet, so could not complete testing. Two men disclosed they were already taking ART (which they did not disclose at the time of receiving the test kit), so did not use the kit.

## 4 DISCUSSION

Offering HIVST kits in community‐based settings successfully reached men in South Africa for HIV testing, including young men, and resulted in 68% linkage to care among men reporting a reactive HIVST. Small cadres of lay health counsellors distributed over 1000 HIVST kits per month to men, of which nearly 12% were used by first‐time testers. Team members were able to provide support for HIVST as needed by clients. These findings support that HIVST strategies found to be efficacious in randomized controlled trials are effective in increasing HIV testing in men in a quasi‐programmatic implementation setting [18, 19]. Taken with the modelling data of community‐based HIVST strategies showing that approaches targeting adult men are among the most cost‐effective testing interventions in high‐HIV prevalence settings like South Africa, these data suggest that HIVST could and should be scaled up country‐wide [20].

In addition to the variety of locations targeted for HIVST distribution, providing men with options for test type (blood‐ and oral‐fluid‐based) and support during testing (demonstration of test kits, staff‐assisted testing/interpretation or no additional support) was valued. We observed differences in the proportion of first‐time HIV testers, prevalence of circumcision and kit type preferences between the two study districts, highlighting the need for options to address preferences in blood versus oral‐based HIVST, locations for testing and support needs among the diverse group of men we tried to reach [21, 22]. Studies have alternately identified populations with strong preferences for blood‐based kits[23] and oral‐fluid‐based kits[24]; our finding that both types were initially selected and 90% of men would test again with the type they chose supports the importance of providing choice of oral fluid and blood‐based HIVST.

First‐time testers, men who elected to test on site, and men who used alcohol heavily were more likely to have a reactive HIVST than repeat testers, men who tested off site and men who had lower alcohol use. These findings are encouraging in showing that HIVST can reach men with these risk factors, and also confirms these risk factors for undetected HIV infection. Alcohol use is a known risk factor for HIV transmission, but many men in South Africa who use alcohol heavily report barriers to accessing HIV services, in some cases related to their alcohol use [25, 26]. HIVST may be a particularly effective component of strategies to provide HIV testing to men who use alcohol heavily.

In all distribution settings, HIVST distribution was acceptable. A small incentive was adequate to promote reporting of HIVST results for over 80% of participants. Without additional incentives to further engage in care, we observed that 68% of men with reactive tests linked to care and initiated ART. This is encouraging, and consistent with community and household‐based HIV testing efforts in the region [27]. We anticipate that a similar rate of linkage could be achieved in a programmatic setting, and thus would not recommend that Departments of Health devote the additional time and resources necessary to ascertain HIVST results. However, this rate of linkage is still well below the target of 90%. HIVST options address some, but not all, barriers men face with HIV testing and linkage. HIVST can be empowering to allow men to test where, when and how they choose, but does not address travel time, costs, lost work and stigma associated with attending public health facilities to initiate treatment, even when ART is free and available.

In this study, men who elected to test on site (with or without direct assistance from a study team member) were more likely to be HIV‐positive, but less likely to link to care compared to men who took HIVST off site to test at home or by themselves. Much of the discrepancy in linkage was explained by important demographic differences in these two groups. However, the discrepancy suggests ways to target interventions to men at higher risk of not linking to care. For example providing on site rapid ART initiation for men with a reactive HIVST and confirmatory test could overcome the initial linkage to care barrier in this population, a service not required for all men needing to link to care, but may benefit this particular at‐risk population. The CASCADE study in Lesotho demonstrated that immediate ART initiation after community‐based HIV testing increased linkage to care and viral suppression compared to referral to clinic facilities to initiate treatment, and an early study of linkage after self‐testing found that home‐based confirmatory testing and ART initiation was associated with greater ART initiation than clinic referral [28, 29]. Additional linkage and support tools, such as enhanced or automated post‐test follow‐up, automated mobile phone‐based applications providing linkage reminders and resources, and after‐hours, geographically flexible ART initiation approaches, may all have a role in improving retention on the HIV cascade of care for men engaging testing through HIVST [30, 31]. Following testing, behavioural economics approaches, such as lottery incentives, can have short‐term impact on linkage and decrease time to ART initiation [15, 32].

### 4.1 Strengths and limitations

Our program had several notable strengths. We conducted HIVST distribution in a pragmatic way designed to be adapted and scaled up to a programmatic setting, where resources for individualized services and follow‐up may be limited. Although our project used additional resources to follow up participants beyond what may be feasible in a programmatic setting, these were limited to ascertainment of outcomes: incentives were provided for participants to report results to assess outcomes, and follow‐up phone calls to assess HIVST use and linkage to care. Because incentives to report results were not contingent on the result itself, we do not anticipate significant bias was introduced as a result of providing the incentives. In summary, we demonstrated the feasibility of rapid, large‐scale distribution of HIVST to reach men, including men who have not yet accessed HIV testing, and high rates of results ascertainment, and linkage‐to‐care.

Our study had some limitations. HIV testing history, HIVST results and linkage to care data were obtained by self‐report, which is subject to responder bias and social desirability bias. For a variety of reasons, people who know their HIV status, including persons on ART, do not always disclose this knowledge in testing settings [33]. There is the possibility that some participants with known HIV‐positive status and already on ART participated in HIVST without disclosing their testing and treatment history; this may have overestimated the true proportion of first‐time testers. However, education was provided at every test distribution session to discourage HIVST among persons already on treatment. HIVST results from on site testing were visually confirmed by study staff and determined reliable interpretation of HIVST results. Additionally, we were able to validate a subset of self‐reports of reactive HIVST results and self‐reports of linkage to care with checking clinic registers, and did not find significant inaccuracy in reporting. Our data on linkage may under‐represent linkage to care and ART initiation, as HIVST‐reactive participants may have initiated ART after the single linkage ascertainment call, which would not have been captured. We were not able to formally assess the cost‐effectiveness of the project, which remains a gap in our understanding of the overall impact of HIVST as part of a broader HIV strategy. Finally, due to the pragmatic nature of this implementation, we were unable to ascertain results of all HIVST kits (19% missing data, including 27% missing data among men who took HIVST kits off site to test) and confirm all linkage data among persons with a positive test (19% missing data). This rate of missing data limits the strength of confidence in the predictors to participants who had complete data available, and may not fully extend to the entire population of men in the study sample, particularly the “hardest‐to‐reach” men whom we were unable to contact. However, we note that less than 20% missing data at each step of the cascade still reflects an overall high rate of retention, from which we can draw important conclusions about the majority of men who we were able to contact, and highlights the real‐life challenges of ascertaining outcomes of self‐testing in programme settings.

## 5 CONCLUSIONS

Men continue to contribute disproportionately to undiagnosed PLHIV in South Africa. HIVST distribution to men in community settings, including workplaces and social venues, is a feasible strategy to increase the number of men learning their HIV status in South Africa. This strategy can reach thousands of men per month with limited staff and resources, though cost‐effectiveness data are lacking and should be urgently pursued as a priority. Essential components of the acceptability of HIVST were access to multiple testing modalities (blood and oral‐fluid‐based HIVST) and availability of staff support. Although HIVST distribution increased the number of men who learned their positive HIV status (“first 90”) and resulted in moderate linkage to ART initiation (approximately 68%), HIVST distribution alone was not sufficient to ensure complete linkage to care. Additional strategies and support are needed to increase engagement of HIV‐infected men in linkage to ART after a reactive HIVST result to optimize the HIV cascade; these should be pursued aggressively in combination with continued access to HIVST.

## COMPETING INTERESTS

The authors declare that they have no competing interests.

## AUTHORS’ CONTRIBUTIONS

AES, CLC, HvR and RVB designed the study. MK, AvH, KS, OK and NS conducted the project and collected the data. TTS and AES analysed the data. AES and RVB drafted the manuscript. All authors contributed to revisions and content of the final manuscript.
